# Data on substantial gravity of carbon dioxide due to pressured metered-dose inhaler steroid treatments for the 2006 year in Japan

**DOI:** 10.1016/j.dib.2018.08.070

**Published:** 2018-09-12

**Authors:** Norihide Murayama, Kikuno Murayama

**Affiliations:** MURAYAMA Pediatrics, 3-2-33 Nagayoshi-Nagahara-Higashi, Hirano-ku, Osaka-shi, Osaka 547-0013, Japan

**Keywords:** HFA, HFC, ICS, pMDI, CO2, Greenhouse gas, Global warming, Montreal protocol, Kyoto Protocol

## Abstract

People all over the world should work in each individual against global warming due to greenhouse gas that is made up of a majority of carbon dioxide. On the other hand chloro-fluoro-carbon (CFC) was used with pressured metered-dose inhaler steroid therapy, but CFC became banning the use because of ozone depleting substance. Hydrofluorocarbon (HFA134a, tetra-fluoro-methane) is used as alternative CFC until now. Less-famously hydro-fluoro-carbon (HFA134a) have 1300-fold (mole ratio) energy of heat-trapping relative to carbon dioxide.

On an extremely localized story, we derived substantial gravity of carbon dioxide from sales total of pressured metered-dose inhaler (pMDI) steroid drugs for the year in Japan. The amount of total sales of inhaled corticosteroid drugs on annual 2006 year was 320 hundred-millions yen. 88 hundred-millions yen (27.4% for total ICS sales) was accounted for pressured metered-dose inhaler steroid. Now in Japan there are three kinds of pressured metered-dose inhaler steroid drugs which all use tetra-fluoro-methane (HFA134a). In fact total gravity of tetra-fluoro-methane (HFA134a) from pressured metered-dose inhaler steroid for annual 2006 year was 19.7 t and substantial gravity of carbon dioxide was 10.8 thousand ton. As total gravity of carbon dioxide production throughout the year in Japan was 13 hundred-million ton. Therefore substantial gravity of carbon dioxide by steroids pressured metered-dose inhaler was very small (0.001%) compared to total carbon dioxide production in Japan. Until today carbon-dioxide reducing make very slow progress, for that reason medical service worker unexceptionally should exert an effort for carbon-dioxide reduction if only slightly through the daily clinical examination.

**Specification table**

**Table:** 

**Subject area**	*Chemistry*
**More specific subject area**	*Global warming*
**Type of data**	*Figures and table*
**How data was acquired**	*Interest in global warming, Drugs sales in Japan 2006,*
	*CHRONOLOGICAL SCIENTIFIC TABLES 2006 Japanese version*
**Data format**	*Images*
**Experimental factors**	*N/A*
**Experimental features**	*N/A*
**Data source location**	*Japan*
**Data accessibility**	*The images are available with this article*

**Value of the data**

• Unique and important data from new stand point.
• pMDI products substantial amount of CO2.
• The reason to select dry powder or solution of three ICS inhalation methods.
• Enlightenment of global warming.

## 1 Data

There are three kinds of method (electric nebulizer, dry powder and pMDI) for inhaled cortico-steroid therapy. All of pMDIs need alternative CFC such as HFC (HFA) 134a that is not Ozone depleting substance but greenhouse gas. More over HFA134a has 1300 folds (mole ratio) efficacy of greenhouse gas compared with CO2. We examined carbon dioxide emissions due to extremely located pMDI from drug sales 2006 in japan. Total sales of ICS in 2006 japan was 3200 hundred million yen. Sales of pMDI consisted in 27.4% (BDP-pMDI; 19% and FP-pMDI; 8.4%). Substantial gravity of carbon dioxide due to only steroid pMDI was 10835 ton/year in Japan. Total gravity of carbon dioxide production throughout the year in Japan was 13 hundred-million ton. Therefore substantial gravity of carbon dioxide by steroids pMDI was very small (0.001%) compared to total carbon dioxide production in Japan. Until today carbon-dioxide reducing make very slow progress, for that reason medical service worker unexceptionally should exert an effort for carbon-dioxide reduction if only slightly through the daily clinical examination. New technique for pMDI is necessary for HFA (CO2) reduction.

## 2 Experimental design, materials and methods

### 2.1 Mechanism of global warning

**Fig. 1:**
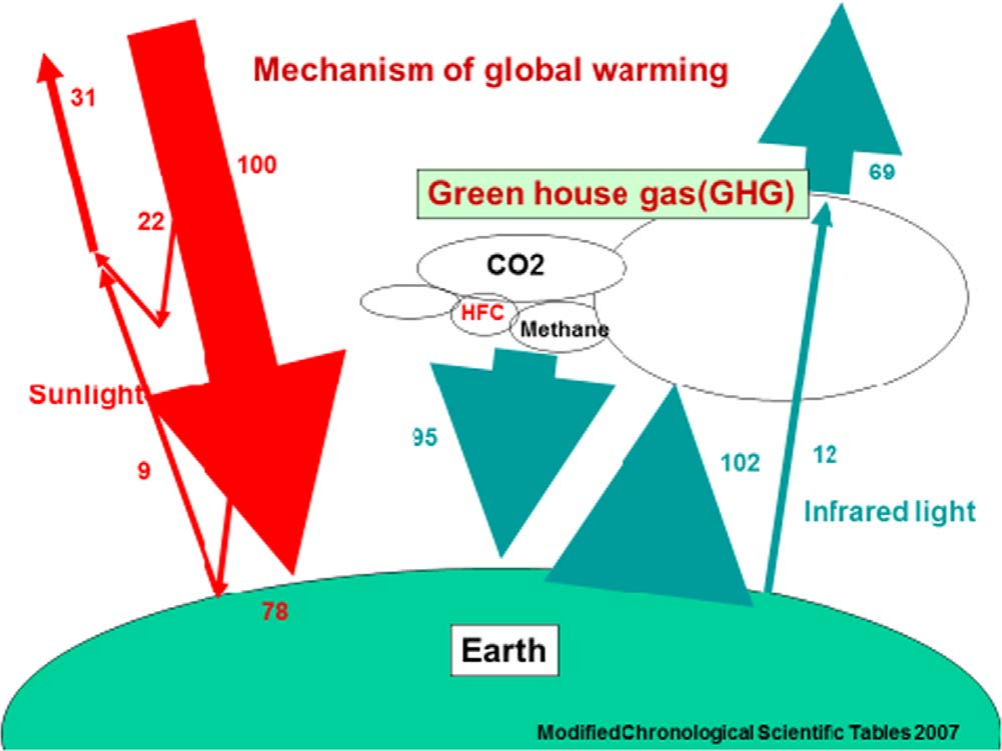
Homeostasis of the Earth.

Homeostasis of the Earth (Fig. 1.).

Fig. 1 shows Homeostasis of the Earth. As amount of 100 light energy light reach to the earth, in result amount 100 of heat energy converted from light energy release to the outside of the earth. But heat energy converted from light energy on the face earth reflex by atmosphere. Repeat heat energy release and reflex give us global warming (Homeostasis) for not only human being but also all live being. But in atmosphere CO2, methane gas, HCF become to increase, reflex of heat energy increase, finally temperature on face earth increase (global warming).

Red the arrows indicate light energy and the blue arrows indicate thermal energy (Fig. 1.). Light energy is exchanged to thermal energy on the face of the earth. A mainly part of light energy is reflected by atmosphere that is reflected by face of the earth. This repeat process keep thermal of the face of the earth called homeostasis. In atmosphere greenhouse gas such as CO2, HFC (Hydro-fluoro-carbon) and etc. are increased, temperature of the face on the earth is up those bring global warming and abnormal weather.

### 2.2 Montreal and Kyoto protocol, Greenhouse gas (Table 1)

**Fig. 2:**
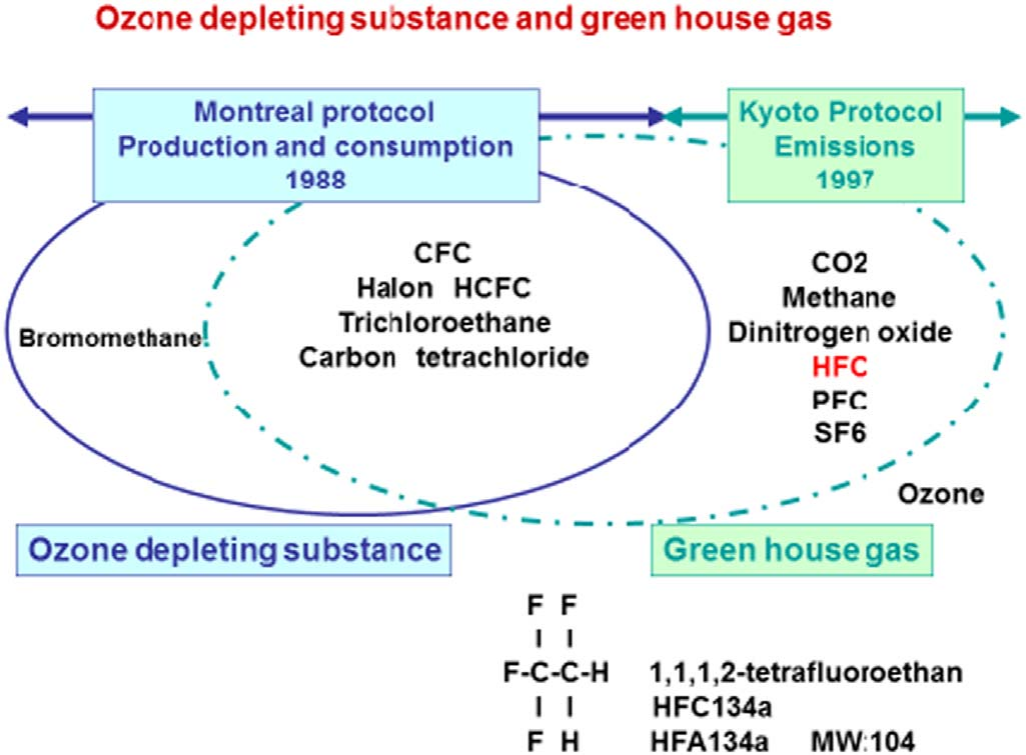
Montreal and Kyoto protocol.

On 1988 Montreal protocol decided to be inhibited Ozone depleting substance product such as CFC (Chloro-fluoro-carbon) (Fig. 2.). On 1997 Kyoto protocol decided to be inhibited Greenhouse gas such as HFA134a (Hydro-fluoro-alkane) or HFC (Hydro-fluoro-carbon) 134a until 2030 (Fig. 2.). HFC134a and HFA134a are same substances of 1, 1, 1, 2-tetrafluoroethane. There are various kinds of greenhouse gas HFA134a belongs to greenhouse gas (Table 1). The amount of HFA134a using pMDI is very few dose but cannot be ignored dose.

**Table 1:** Greenhouse gas(GHG).

Substance	Use	Ozone depleting index	Global warming index
Ozone depleting substance			
CFC	Refrigerator, air con, Insulation	0.6–1.0	4600–14,000
		(CFC12:1.0)	(CFC12:10,600)
HCFC	Refrigerator, air con, Insulation	0.001–0.52	120–2400
		(HCFC22: 0.055)	(HFCFC22: 1700)
Halon	Extinguishing	3.0–10.0	

Alternatives for chlorofluorocarbon			
HFC	Refrigerator, Air con, pMDI	0	140–11,700
			(HFC134a: 1300)
PFC	Cleaning agent, Semiconductor	0	6500–9200
			6500
SF6	Electric insulating, Semiconductor	0	23,900
		

CFC: Chlorofluorocarbon, HCFC: Hydrochlorofluorocarbon, HFC: Hydrofluorocarbon, PFC: Perfluorocarbon, SF6: Sulfur hexafluoride.

### 2.3 2006 Current ICS drugs (Table 2)

By big classification there are three kinds of ICS. Only pMDI needs HFA134a. In 2006 pMDI there are 7 kinds of pMDI (Table 2), BDP (50, 100 μg/puff) (Dainippon Sumitomo Pharma Co., Ltd), FP (50, 100 μg/puff) (GlaxoSmithKline K.K) and Ciclesonide (50, 100, 200 μg/puff) (TEIJIN PHARMA LIMITED).

**Table 2:** Current drugs of inhaled corticosteroids in 2006 Japan.

Methods	Drugs being sold
pMDI	BDP (50, 100 μg/puff) Dainippon Sumitomo Pharma Co., Ltd
	FP (50, 100 μg/puff) GlaxoSmithKline K.K.
	Ciclesonide (50, 100, 200 μg/puff) TEIJIN PHARMA LIMITED
DPI	FP Diskus (50, 100, 200 μg/blister) GlaxoSmithKline K.K.
	Rota-disk (50, 100, 200 μg/blister) GlaxoSmithKline K.K.
	BUD (100, 200 μ/blister) AstraZeneca
Suspension	BUD (250, 500 μg/ampule) AstraZeneca
DPI (Combination of drugs)	FP(100, 250, 500 μg/blister)+LABA, GlaxoSmithKline K.K.

### 2.4 Contents of pMDI (Table 3)

Each can of pMDI includes HFA134a, BDP (50, 100 μg/puff); 7.8 g/can, FP (50 μg/puff); 10.6 g/can, FP (100 μg/puff); 7.0 g/can, Ciclesonide (50, 100 μg/puff); 5.9 g/can, Ciclesonide (200 μg/puff); 3.0 g respectively.

### 2.5 Sale share of ICS (Fig. 3)

**Fig. 3:**
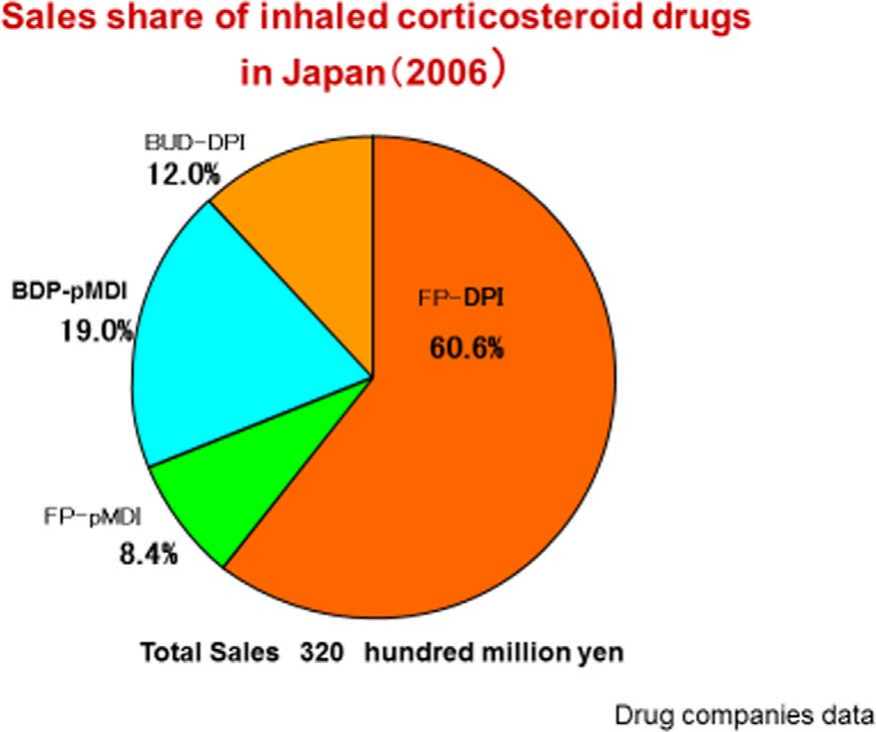
Private data of Japan Pharmacists Association.

Total sales of 2006 ICS was 320 hundred million yen (Fig. 3). In it pMDI occupied 27.4% (BDP-pMDI; 19% and FP-pMDI; 8.4%) (Fig. 3). When we calculate the number of can, we selected high price of can on occasion (Table 3). Because from ICS sale data, on BDP-pMDI we could not distinguish 50 μg-can or 100 μg-can (Table 3).

**Table 3:** Current drugs of pMDI corticosteroids 2006 in Japan.

pMDI (Contents)	Content WT	HFA WT	puff/can times	HFA WT/puff(mg)	Price/can (yen/can)	Price/HFA1g (yen/g)	HFA(mg)/MDI 200 μg
BDP (HFA, Ethanol and BDP)							
50 μg/puff	8.7 g	7.8 g	100	78	3023	388	312
100 μg/puff	8.7 g	7.8 g	100	78	4024	516	156

FP (HFA,FP)							
50 μg/puff	10.6 g	10.6 g	120	88	2367	223	353
100 μg/puff	7.0 g	7.0 g	60	117	2378	340	233

Ciclesonide (HFA, Ethanol, Ciclesonide)							
50 μg/puff	6.6 g	5.9 g	112	53	1814	307	210
100 μg/puff	6.6 g	5.9 g	112	53	2380	403	105
200 μg/puff	3.3 g	3.0 g	56	54	2380	403	54

### 2.6 Substantial gravity of carbon dioxide

Substantial gravity of carbon dioxide was calculated by below formula.

Substantial gravity of carbon dioxide = sum total (HFA gravity/can × sale number of can × CO2 molar weight/ HFA134a molar weight × 1300).

### 2.7 Substantial gravity of carbon dioxide (Table 4)

Substantial gravity of carbon dioxide from BDP, FP and Ciclesonide were 6490, 4345 and 0 t respectively. Because Ciclesonide started to sale on 2006, there is no data of ciclesonide sale. Substantial gravity of carbon dioxide due to steroid pMDI was10835 ton/2006 year in Japan (Table 4). Total gravity of carbon dioxide production throughout the year in Japan was 13 hundred-million ton. Therefore substantial gravity of carbon dioxide by steroids pMDI was very small (0.001%) compared to total carbon dioxide production in Japan. The amount is bigger than we expected. It is not until existence of warming gas by steroids pMDI for asthmatic treatment.

**Table 4:** Substantial gravity of carbon dioxide by steroid pMDI in Japan (2006) Carbon dioxide weight = HFA weight × 1300 × 44(CO2,MW)/104(HFA,MW) HFA weight(g) = Amount of sales/price(can) × HFA content(g/can).

	Amount of sales hundred million (yen)	Price of can (yen)	Quantity of can thousand	HFA weight (ton)	Carbon dioxide weight(ton)
BDP	60.8	4024	1510	11.8	6490
FP	26.9	2378	1131	7.9	4345
Total	87.7		2641	19.7	10835

Total carbon dioxide weight due to HFA of pMDI for one year accounts approximately the weight of ten thousand compact auto car.

Until today carbon-dioxide reducing make very slow progress, for that reason medical service worker unexceptionally should exert an effort for carbon-dioxide reduction if only slightly through the daily clinical examination.

